# Mucosal Applications of Poloxamer 407-Based Hydrogels: An Overview

**DOI:** 10.3390/pharmaceutics10030159

**Published:** 2018-09-12

**Authors:** Elena Giuliano, Donatella Paolino, Massimo Fresta, Donato Cosco

**Affiliations:** 1 Department of Health Sciences, University “Magna Græcia” of Catanzaro, Campus Universitario “S. Venuta”, Viale S. Venuta, I-88100 Catanzaro, Italy; giuliano.ele@gmail.com (E.G.); fresta@unicz.it (M.F.); 2 Department of Experimental and Clinical Medicine, University “Magna Græcia” of Catanzaro, Campus Universitario “S. Venuta”, Viale S. Venuta, I-88100 Catanzaro, Italy; paolino@unicz.it

**Keywords:** poloxamer 407, bioadhesion, mucosal drug delivery, hydrogels, thermo-reversible features

## Abstract

Poloxamer 407, also known by the trademark Pluronic^®^ F127, is a water-soluble, non-ionic triblock copolymer that is made up of a hydrophobic residue of polyoxypropylene (POP) between the two hydrophilic units of polyoxyethylene (POE). Poloxamer 407-based hydrogels exhibit an interesting reversible thermal characteristic. That is, they are liquid at room temperature, but they assume a gel form when administered at body temperature, which makes them attractive candidates as pharmaceutical drug carriers. These systems have been widely investigated in the development of mucoadhesive formulations because they do not irritate the mucosal membranes. Based on these mucoadhesive properties, a simple administration into a specific compartment should maintain the required drug concentration in situ for a prolonged period of time, decreasing the necessary dosages and side effects. Their main limitations are their modest mechanical strength and, notwithstanding their bioadhesive properties, their tendency to succumb to rapid elimination in physiological media. Various technological approaches have been investigated in the attempt to modulate these properties. This review focuses on the application of poloxamer 407-based hydrogels for mucosal drug delivery with particular attention being paid to the latest published works.

## 1. Introduction

Hydrogels are polymeric materials that are characterized by a three-dimensional network that can retain a large amount of water or biological fluid under physiological conditions; they can be used as delivery systems due to the unique properties of sol–gel conversion that is modulated by a specific biological stimulus [1]. Some systems are in a liquid state before administration into the body, while a sol-to-gel transition occurs after the injection. Thanks to this useful property, these systems offer several advantages, namely an easy preparation procedure due to their liquid-like behavior at room temperature, then gelation following the administration accompanied by a prolonged residence time at the application site, and a sustained drug release.

The following mechanisms modulate the formation of the gel: the formation of ionic cross-links, changes in pH and temperature, plus UV irradiation [2]. This in situ thermo-gelling has no need for external agents, because the gelation occurs when the temperature increases, as is true in the case of the administration into the body [3]. This is why pharmaceutical research has been focusing its attention over the last few decades on the development of thermo-sensitive hydrogels for various applications [4].

Poloxamers are thermo-responsive polymers that are widely used in the development of in situ gel systems. These polymers are ABA-type triblock copolymers that are composed of polyoxyethylene (A) and polyoxypropylene (B) units. Due to their chemical structure, they are characterized by an amphiphilic nature, which makes them useful surfactants that are employed in many industrial fields [5]. Among these, poloxamer 407 (P407) is used for its good solubilizing capacity, low toxicity, good drug-release characteristics, and its compatibility with numerous biomolecules and chemical excipients [6]. P407 thermo-responsive hydrogels are widely investigated as useful mucosal drug delivery systems, due to their non-irritating action on the biomembranes, and due to the opportunities that they afford in delivering drugs to a specific compartment, maintaining the required concentration for a prolonged period of time, and decreasing the efficacious dosage and side effects [7,8].

These molecules are able to form entanglements or non-covalent bonds with mucus, thus favoring a great degree of interaction with various biological tissues, prolonging the residence time of the formulation at the application site.

In this review, the use of poloxamer 407-based hydrogels for the development of mucoadhesive drug delivery systems will be described, with specific focus on the most important applications from recent years.

## 2. Bioadhesion and Mucoadhesion

### 2.1. Theories and Mechanism

Bioadhesion is generally defined as the interaction between two materials (at least one of which is a biological substrate) for a given period through interfacial forces, with a consequent decrease in the surface energy of the system [9]. If the substrate is a mucous layer, the term mucoadhesion is preferred to define the interesting interaction that comes about at the interface between a pharmaceutical dosage form and the biomembranes [10]. It is a complex multifactorial process and numerous theories have been proposed, in order to explain the various mechanisms involved [11,12,13,14,15,16,17,18].

None of them can wholly explain the mucoadhesion phenomenon [19]. It is probably brought about by the synergistic combination of the different mechanisms: firstly, adhesive materials do wet and swell (wetting theory), then non-covalent bonds with mucus occur (as advanced by the adsorption theory) and, finally, they penetrate into the tissues or into the surface of the mucus membrane (diffusion theory), causing fractures in the layers, or electronic transfer, or simple adsorption phenomenon, which finally leads to effective mucoadhesion (fracture, electronic, and adsorption theories) [10,20,21].

In a more simplified theory, the mucoadhesive mechanism is generally reduced to two steps: the contact phase, and the consolidation stage [13,22,23]. The first is characterized by contact between the bioadhesive molecule and the mucosal membrane, while in the second, the materials cause to non-covalent interactions such as hydrogen bonds and Van der Waals forces [17,21,24]. This can be explained by the following two hypotheses: the diffusion theory affirms that mucoadhesive molecules characterized by the chemical residues that are able to form hydrogen bonds (–OH, –COOH) can bind the glycoproteins of the mucus, diffusing into the various layers [17,21,25]; the dehydration theory postulates that the materials capable of gelling in aqueous environments can leak their water, due to a difference in osmotic pressure from that of mucus [26]. This process can increase the contact time with the mucosal membrane and favor mixing [16].

### 2.2. Mucoadhesive Polymers

Mucoadhesive systems have great potential. As previously described, the increased residence time at the application site can provide a controlled, sustained drug release, decreasing the number of administrations and increasing patient compliance [27,28].

Mucoadhesive polymers are usually macromolecules that are characterized by numerous hydrophilic residues that are capable of binding glycoproteins and giving rise to hydrocolloids.

In pharmaceutical application, their use favors the modulation of the bioavailability, absorption, and delivery of drugs, while decreasing their side effects. An ideal polymer should (1) easily retain hydrophilic and lipophilic drugs, and not hinder their release, (2) preferably form non-covalent bonds with mucin, (3) inhibit the action of local enzymes and promote drug absorption, (4) adhere as quickly as possible to biological tissues, (5) be atoxic, and (6) be low-cost [11,29].

The interaction between the bioadhesive polymer and the mucin that composes the mucous layer is influenced by various parameters such as hydrogen bonding, the occurrence of anionic or cationic electrostatic interaction, high polymeric molecular weight, polymeric concentration, chain flexibility, and surface energy properties [8,30,31,32,33].

Mucoadhesive polymers can be either natural or synthetic molecules, they can possess different surface charges, and they can be classified as conventional polymers (first generation) or novel material (second generation) [34]. The first generation-type polymers can be divided into three categories: cationic, anionic, and non-ionic compounds [13]. They are composed of hydrophilic molecules containing functional polar groups, i.e., hydroxyl, carboxyl, and amine, which are able to form hydrogen bonds [13,16]. Chitosan, cellulose, carbomer, alginate, and their derivatives all belong to this class [34,35,36,37,38,39].

The advantage of conventional polymers is related to their natural origin; however, they form non-covalent and non-specific bonds with the mucus, consequently providing mucoadhesive bonds that are too weak to assure prolonged retention times with the mucus and oppose the fast elimination promoted by normal mucus turnover [40].

The novel material or second-generation mucoadhesive polymers are multifunctional materials that were developed in the late 90s, which differ from conventional materials because they are able to form both covalent, and non-covalent bonds with the mucus, thus inducing strong chemical interaction. Lectins and thiolated polymers are typical examples [41,42,43,44].

The development of the second-generation of polymers was an important turning point in mucoadhesive research, but the use of these new materials brought up new problems. In fact, they need to be chemically modified through a synthetic process and their potential cytotoxicity needs to be evaluated, as well as oxidation and stability features [12,45,46].

## 3. Poloxamers

### 3.1. General Characteristics and Proprieties

Poloxamers, also known under various trademarks as Pluronics^®^, Synperonics^®^, or Lutrol^®^, are a class of water-soluble non-ionic triblock copolymers consisting of a hydrophobic core of polyoxypropylene (POP) between two hydrophilic units of polyoxyethylene (POE) [5,47,48].

Their specific characteristics depend on the lengths of the chains of the various blocks. Indeed, poloxamers are available in different molecular weights and physical forms that are related to the POP:POE ratio. Due to their chemical structure, they are characterized by an amphiphilic nature that makes them useful surfactants, stabilizers, solubilizing, and coating agents [5,49,50].

A nomenclature is adopted in order to provide useful information about the physico-chemical properties of the various derivatives, according to which each copolymer is characterized by three numbers representing the molecular weight of the hydrophobic portion, and the amount of the hydrophilic chains. The first two digits, multiplied by 100, provide the average molecular mass of the POP section, while the percentage amount of POE is obtained multiplying the last digit by 10 [5]. In the case of commercial formulations, the physical state is specified by a consonant (P: Paste, F: Flake, L: Liquid) followed by two or three digits: in this case, the first digit (or the first two digits), multiplied by 300, indicates the approximate molecular weight of the hydrophobic block while the last one, multiplied by 10, represents the percentage of POE content. For example, poloxamer 188 (or Pluronic^®^ F68) is characterized by a POP average molecular weight of 1800 Da, and by approximately 80% of the POE [51].

**Figure 1:**
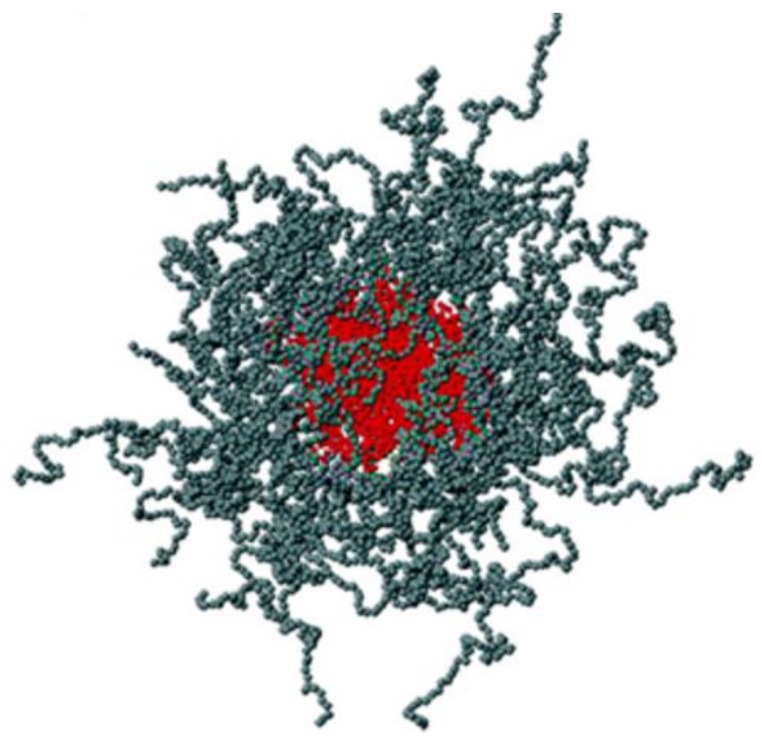
Schematic representation of poloxamer 407-micelles obtained by molecular dynamics in the CGIS (coarse-grained implicit solvent) model. In the CGIS model, water is treated as an implicit viscous solvent, while polymer chains are represented as bead-spring polymers, where each bead represents an oxyethylene (OE) or a oxypropylene (OP) monomer. The micelle core is made up of polyoxypropylene (POP) blocks, while the corona is formed by the polyoxyethylene (POE) blocks. Reprinted from [56] with permission from American Chemical Society, 2006.

A peculiar feature of these polymers is their thermo-gelling property, due to their capacity to self-assemble into micelles in an aqueous solution (Figure 1). This phenomenon was extensively studied by Alexandridis and co-workers in the 1990s [52]. The micellization process depends on two key parameters: the critical micelle concentration (CMC); that is, the copolymer concentration that is required to obtain the micelles, and the critical micelle temperature (CMT). Individual block copolymer molecules, often called unimers, form a solution in water below the CMC, while above this value, aggregation phenomena occur [52,53]. The CMC value is inversely proportional to the number of POP units, indicating that the micellization process is mainly a function of the hydrophobic chain [54]. The CMC of poloxamers decreases with increasing temperature as a consequence of the difference in the solvation of the POE and POP blocks, thus minimizing interaction with the solvent; this means that poloxamers are characterized by a specific CMT [55,56].

Micellization is the first step of gelation because the physical packaging of the micellar structures is fundamental. The gelling phenomenon is reversible and it is related to a sol-gel transition temperature (T_sol-gel_): below this value, the sample is in a liquid form, while above it, the solution becomes solid-like.

Poloxamers are GRAS (generally recognized as safe) excipients, and they have been amply used in the development of many pharmaceutical formulations, and proposed for various applications (injectable, oral, rectal, ophthalmic, cutaneous, nasal, and vaginal systems) ranging from the targeting of the central nervous system, to drug delivery, gene therapy, tissue engineering, and diagnostics [51,57,58,59,60].

### 3.2. Poloxamer 407

Poloxamer 407, principally known by the brand name Pluronic F127, is a copolymer characterized by a molecular weight of around 12.6 kDa (POE101 POP56 POE101) and it contains ~70% of polyoxyethylene, which contributes to its hydrophilicity [61]. Poloxamer 407 is an excipient of various formulations that is approved by the U.S. Food and Drug Administration (FDA) for pharmaceutical application [6,61,62].

It is a non-ionic surfactant, having a good solubilizing capacity, low toxicity, good drug release characteristics, and compatibility with cells, body fluids, and a wide range of chemicals [6,63]. All of these features make it a useful compound with which to develop various pharmaceutical formulations [60]. For example, Famciclovir (Apotex Inc. Toronto, ON, Canada), Gabapentin (Glenmark Generics Inc., Mahwah, NJ, USA), Isentress (Merck & Company Inc., Whitehouse Station, NJ, USA), Multaq (Sanofi-Aventis, Paris, France), Neurontin (Pfizer U.S. Pharmaceuticals Group, New York, NY, USA), and Omeprazole (Dr. Reddy’s Laboratories Inc., Beverley, England, UK) are solid state forms containing the copolymer.

Poloxamer 407 has been used as a detergent, surfactant, and stabilizer. It facilitates the solubilization of hydrophobic molecules, promoting their rapid and complete dissolution in polar media [6,47]. For example, the solubility of piroxicam and nifedipine in water increased by 11- and 27-fold, respectively, after the addition of Poloxamer 407 [64,65].

**Figure 2:**
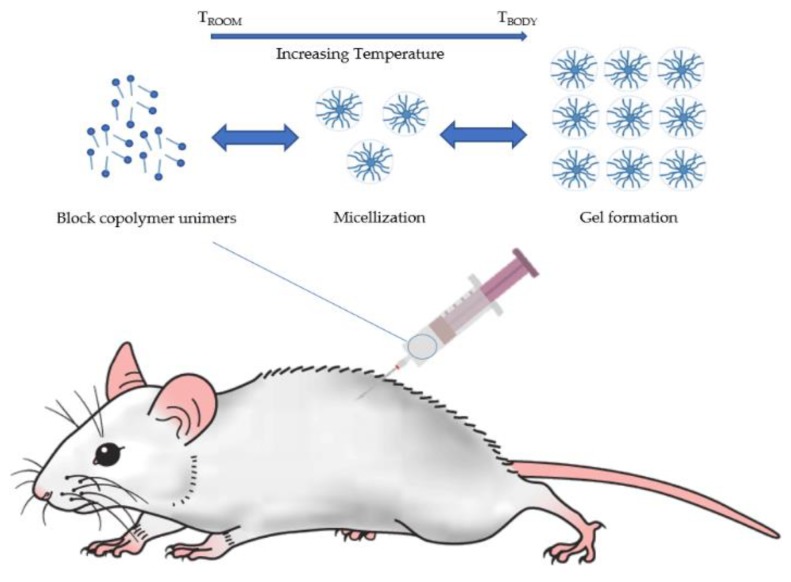
Schematic illustration of the in situ gelation mechanism of a thermo-responsive P407 aqueous solution. Upon a temperature rise, a breakage of the hydrogen bond is established between the aqueous solvent and the copolymer hydrophilic chains. Desolvation induces the hydrophobic interaction among the POP blocks, the formation of spherical micelles and, successively, the gelation process.

Aqueous solutions containing poloxamer 407 have interesting, reversible thermal characteristics. As previously described, the molecules of poloxamer 407 are surrounded by a hydration layer at low temperatures, while an increase in temperature induces a breakage of the hydrogen bond between the aqueous solvent and the hydrophilic chains of the copolymer. This desolvation favors hydrophobic interaction among the POP blocks, and the formation of spherical micelles. The gelation process is promoted by this phenomenon (Figure 2).

A temperature increase initially induces the rearrangement of poloxamer 407-based micelles into a cubic structure, and then favors a hexagonal configuration [60].

The formulations containing the copolymer at a concentration of 15–30% *w*/*w* are characterized by gelation at body temperature. Poloxamer 407-based thermo-sensitive hydrogels have been used for the delivery of active compounds characterized by different physico-chemical properties, with the aim of obtaining a controlled release.

Ricci et al. investigated the application of poloxamer 407 formulations (with a polymer concentration of 20, 25 and 30% *w*/*w*) for the entrapment and sustained release of lidocaine, a local anesthetic that is used to treat acute and chronic pain, which is characterized by the short duration of its pharmacological effects [66,67]. They demonstrated that the increase of the copolymer concentration and the gel viscosity decreased the drug release rate and the gel dissolution time, prolonging the effects of the drug. The influence of additives to the poloxamer solution, such as polyethylene glycol 400 or inorganic salts, was also shown; they induced an increase in the drug release and diffusion coefficients [66,67]. In addition, Xuan et al. used poloxamer 407 for the intramuscular delivery of piroxicam, another poorly water-soluble compound with a brief duration used in the management of chronic pain. They developed an easily administered, biodegradable and biocompatible injectable hydrogel that gelled rapidly in the body and favored a release of the drug for a long time [68]. Bansal and co-workers formulated and characterized an in situ gel containing two antibiotics—levofloxacin and metronidazole—to obtain the controlled release of these two drugs in the treatment of periodontitis. They used a 20% (*w*/*v*) solution of the co-polymer, with the addition of chitosan (1.5% *w*/*v*), obtaining a syringeable, thermo-responsive, mucoadhesive gel that allowed a prolonged, controlled release of the bioactives, together with significant pharmacological efficacy against a broad range of microbes [69].

Moreover, the poloxamer 407-based hydrogels have been used with or without other additives, as a delivery system for proteins, such as interleukins, insulin, or bovine serum albumin, in order to prolong their half-lives and optimize their therapeutic effects [70]. Many experimental investigations have shown that these formulations are characterized by a great thermo-stability that favors the structural integrity of the entrapped proteins [57,71].

Because of the easy administration and the drug release characteristics, thermo-sensitive gels are being widely employed in topical administration. In the case of dermal application, there are numerous studies that are focused on the delivery of analgesic or anti-inflammatory drugs for the treatment of local pain and inflammation [60,72]. The topical administration of poloxamer 407-hydrogels has also been evaluated for the delivery of anticancer agents, antibiotics, and anti-diuretics and antiseptics [60].

Despite its multiple benefits, poloxamer 407 is characterized by a short residence time, due to its rapid dissolution in aqueous media or biological fluids [57,73]. This characteristic is a critical challenge to be met, and it could be overcome by the addition of specific compounds, such as other mucoadhesive polymers or ionic strength- and pH-controlling agents [74,75,76].

Besides acting as a carrier for drug delivery, poloxamer 407 has clinical and therapeutic uses in the treatment of various physio-pathological conditions. It can directly translocate into cells, modulating various cellular pathways, mitochondrial respiration, apoptotic signal transduction, the activity of drug efflux transporters, and gene expression [48].

### 3.3. Preparation of Thermo-Reversible Hydrogels

Poloxamer 407 aqueous solutions can be prepared by two methods; in both, the solubilization of the polymer in the aqueous phase is the starting point, but in the “cold” procedure, the dissolution is performed at 4 °C until a clear solution is formed, while in the “hot” method, the aqueous phase is heated to 70–90 °C, and after a complete dissolution of the polymer, the formulation is cooled to favor the gelling process. The dissolution of the polymer takes place under continuous and slow stirring in order to avoid the formation of foam [5,77]. Pereira et al. used different stirring techniques to disperse the poloxamer in water (18% *w*/*w*), with the aim of evaluating the influence of the method of preparation on the T_sol-gel_. They demonstrated that manual and mechanical stirring were not significantly different, while high-performance stirring by an Ultraturrax^®^ (IKA^®^-Werke GmbH & Co. KG, Staufen, Germany) decreased the T_sol-gel_ of the samples. Magnetic stirring is preferred in most experimental investigations, and in industry [78]. Both hot and cold procedures result in a transparent, colorless gel with excellent rheological properties. Generally, the cold method is preferred, due to the increased solvation of the copolymer, which precludes the formation of hydrogen bonds and sample alteration [5,6,77]. This method allows a more manageable sample preparation because, it is easy to prepare P407 solutions at 20–30% (*w*/*w*) characterized by a liquid state at a temperature of 4–5 °C; the solution becomes a gel at room temperature [6]. Various active compounds can be entrapped within the described formulations, obtaining innovative delivery systems [76,79,80,81,82,83,84,85].

The sterilization appears to be compatible and does not significantly influence the viscosity of poloxamer 407-formulations [5,6,86]. Therefore, it is possible to prepare a sterile P407-based hydrogel to be administered by the means of ophthalmic application or by injection [86,87,88].

### 3.4. Measurement of Sol–Gel Transition Temperature and Gelation Time

P407 aqueous solutions show a temperature-dependent liquid–gel transition which is an interesting property for the development of mucoadhesive formulations.

The sol-gel transition temperature is the temperature at which this phenomenon occurs. An optimal gelation temperature of a mucoadhesive formulation should be in the range between 30–36 °C, because a gelation temperature that lower than 30 °C induces the formation of the gel at room temperature causing difficulty in manufacturing, handling, and administering, while a gelation temperature that is higher than 37 °C means that the formulation exists in the liquid state at body temperature, resulting in a rapid elimination after administration [79,89,90].

Therefore, the evaluation of the sol–gel transition temperature is a fundamental prerequisite for the development of an effective mucoadhesive formulation.

The sol-gel transition temperature can also be easily determined by the “magnetic stirrer method”, which is described in various experimental investigations [79,81,90]. Namely, the poloxamer solution is loaded into a transparent vial containing a magnetic bar in a thermostatic water bath at low temperature, and a digital thermo-sensor is immersed into the formulation. The sample is gradually heated while being continuously stirred. When the magnetic bar stops moving due to gelation, the temperature displayed on the thermo-sensor is identified as the gelation temperature [79,90,91].

Another approach is based on rheological analysis using a rotational rheometer. The gelation temperature is evaluated by oscillation measurements performed at a constant frequency value (0.01 and 1 Hz are the most commonly used) and in a temperature range that includes the physiological temperature. In this case, the sol-gel transition temperature will be defined as the point where the elastic modulus (G′) and viscous modulus (G″) curves intersect each other. This is the temperature at which the sample exhibits a switch from a prevalently viscous behavior (G″ > G′) to a prevalently elastic one (G′ > G″) [80,90]. G′ describes the elasticity or the energy stored in the material during deformation, while G″ represents the viscous character or the energy dissipated. These parameters are used to define the rheological characteristics of a sample, i.e., elastic solids, viscous fluids, or viscoelastic materials [80,92].

Some authors prefer to define T_sol-gel_ as the point at which the viscosity is halfway between a solution and a gel [3,82,89,93].

Baloglu et al. used both methods in their study to evaluate the T_sol-gel_ of P407-aqueous solutions with different polymeric concentrations, and the results were compared. According to the rheological method, the sol–gel transition temperatures of the formulations were found to be higher than the results obtained from the magnetically stirred method. The authors explained this difference in a discussion of the theory of the two methods: one based on the subjective observation of the immobilized magnetic bar, the other one was based on instrumental detection, which circumvented subjectivity [90].

Gelation time is an important factor that can also be evaluated by rheological measurements. It is defined as the time needed by the elasticity modulus to become higher than the viscous modulus. A short gelation time at body temperature means a rapid gelation of the formulation after administration, and this is fundamental for preventing its removal from the injection site, prolonging the retention of the active substance in situ. It is determined by applying a constant shear stress to the sample at T_sol-gel_ [3,89].

## 4. Poloxamer 407-Based Mucoadhesive Formulations

Poloxamer thermo-responsive gels have numerous biopharmaceutical applications. In recent years, poloxamer 407 has been particularly commonly employed in mucosal drug delivery because of its thermo-reversible characteristics at physiological temperature [8]. Moreover, the co-polymer does not damage mucosal membranes [94].

In the development of an in situ gel system, the bioadhesive force and the gel strength are crucial factors to be modulated, in order to promote the retention of the formulation at the application site [76,79]. Table 1 describes various formulations of poloxamer 407-based hydrogels with mucoadhesive properties.

The sol-gel transition temperature can be modified by the presence of additives that can modulate the micellization of a co-polymer. Some of these (e.g., sodium chloride, sodium monohydrogen phosphate and sodium dihydrogen phosphate) decrease the T_sol-gel_, due to the formation of strong cross-linking bonds with the poloxamer molecules; others, like hydrochloric acid, form weak hydrogen bonds and increase the gelation temperature [76,80,90].

Other compounds associated with poloxamer 407 are polyvinylpyrrolidone (PVP, a cationic and water-soluble polymer), hydroxypropylcellulose (HPC), and hydroxypropylmethylcellulose (HMPC), carbopol^®^, and polycarbophil (bioadhesive polyacrylic acid derivatives) [113].

Poloxamer 407 is often used in association with other poloxamers, especially poloxamer 188, in order to modulate the T_sol-gel_. Poloxamer 188 is approximately 80% POE and 20% POP units. It is used as an emulsifier, solubilizer, and a dispersing and wetting agent, similar to poloxamer 407. Choi et al. developed in situ-gelling and mucoadhesive liquid suppositories containing acetaminophen, using varying percentages (*w*/*w*) of poloxamers 407 and 188 [79].

**Figure 3:**
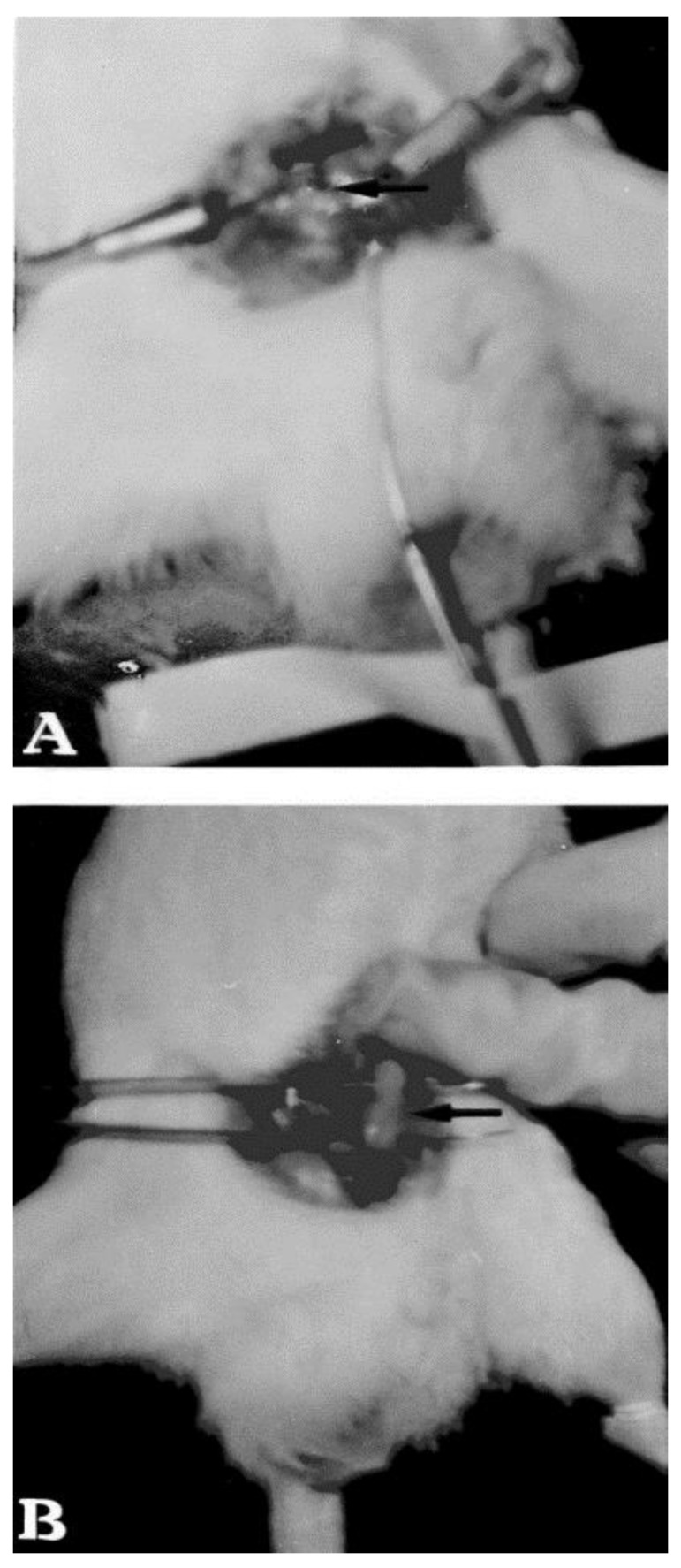
In vivo localization of a liquid suppository in the rectum. Liquid suppository made up of P407/P188/polycarbophil/acetaminophen (15/19/0.8/2.5% *w*/*v*) with 0.1% blue lake was introduced into the rectum of a rat. At 5 min (**A**) and 6 h (**B**) after administration, the rectum was sectioned. Reprinted from [79] with permission from Elsevier, 1998.

Their study demonstrated that the mixture of P407 (15% *w*/*w*)/P188 (15% and 20% *w*/*w*) is optimal for obtaining a suitable gelation temperature. Polymers such as PVP, HMPC, HPC, carbopol and polycarbophil have been used to modulate the gel strength and bioadhesive forces of liquid suppositories containing acetaminophen. The entrapment of the drug induced a slight increase in the gelation temperature of P407/P188 solutions, while PVP, HMPC, HPC did not significantly influence this parameter. On the contrary, the use of carbopol and polycarbophil favored a decrease of the gelation temperature, and notably enhanced (at a concentration of less than 1% *w*/*w*) both the gel strength and the bioadhesive force, preventing the elimination of the suppositories from the rectum [79] (Figure 3).

The same research team investigated the effect of additives on the physicochemical properties (gelation temperature, gel strength, bioadhesive force) of P407/P188 liquid suppositories. In detail, the influence of ethanol, propylene glycol, and glycerine as solvents, of sodium chloride as an ionic strength-controlling agent, and of pH-controlling agents such as hydrochloric acid, sodium monohydrogen phosphate, and sodium dihydrogen phosphate was evaluated. Glycerin, sodium chloride, sodium monohydrogen phosphate, and sodium dihydrogen phosphate (1%, *w*/*w*) were shown to promote the formation of strong cross-linking bonds with the poloxamers, causing a ~60-fold increase in gel strength and 10-fold more bioadhesive force, with a slight decrease in the gelation temperature. Conversely, the use of ethanol, propylene glycol, and hydrochloric acid increased the T_sol-gel_, and slightly decreased the gel strength and the bioadhesive force, due to the weak hydrogen bonds that were formed [76].

Alginate is another biopolymer that is implemented because of its adhesive features. Ryu et al. prepared poloxamer liquid suppositories by adding different mucoadhesive polymers, such as HPC, PVP, carbopol, polycarbophil, and sodium alginate. The latter compound provided the best mucoadhesive force to the formulation, while causing no irritation of the rectal mucosa [95]. In fact, alginate is perfectly compatible with poloxamer 407, and they can both be used to obtain a strong, useful thermo-sensitive gel [114]. A phenomenon of physical instability was observed when small amounts of HPMC were added to poloxamer 407; the sample separated into two phases. This can be remedied by adding propanediol-1,2 to the system [75].

Park et al. investigated the effect of sodium chloride on the release, safety, and rectal absorption of diclofenac sodium delivered by poloxamer gels in rats. Sodium chloride improved the gel strength and did not cause any morphological damage of the rectal tissues at concentrations of less than 0.8% (*w*/*w*) [96].

Gelrite^®^ (deacetylated gellan gum cations), a naturally-derived gelling polymer, showed a positive effect on the bioadhesive properties of thermo-sensitive ophthalmic hydrogels made up of poloxamer P407 containing moxifloxacin hydrochloride, and it also had a positive effect on the modulation of the drug release rate [100].

Several studies have focused their attention on the modulation of the bioadhesive properties of poloxamer 407-gels by means of chitosan, a natural polysaccharide derived from the chitin of the exoskeletons of shrimp and crabs, which is widely used in pharmaceutical application [59,115,116]. The addition of chitosan improved the mucoadhesive characteristics and the gel strength of poloxamer-gels, due to the positively-charged amine residues of the compound, which promote a great deal of interaction with the negatively-charged mucous [101]. Gratieri et al. developed an ophthalmic delivery system for the treatment of ocular diseases, characterized by suitable mechanical and mucoadhesive properties with the aim of prolonging the retention time in the eye. The results showed that chitosan improved the mechanical strength and the texture of poloxamer 407-formulations preserving the mucoadhesive features [101].

Hyaluronic acid (HA) is another biocompatible and biodegradable natural polysaccharide that is used in various biomedical and pharmaceutical applications [7,117,118,119]. Mayol et al. formulated a gel made up of poloxamer 407/HA in order to favor the ocular administration of acyclovir [80]. The addition of HA (150 kDa) did not hamper the thermo-sensitive self-assembling process of poloxamer, and it caused the reinforcement of the gel structure, promoted by the hydrogen bonds between the two polymers. Mucoadhesion tests showed much interaction between the obtained gel and mucin promoted by the synergic effect of the two compounds which made a prolonged and controlled release of acyclovir possible [80].

Small molecules and peptides do not efficiently cross a mucosal membrane, due to their molecular size, poor lipophilicity, or enzymatic degradation. To overcome these complications, one of the most important strategies frequently adopted to bypass the mucosal bilayers is the use of absorption enhancers such as surfactants, bile salts, fatty acid salts, phospholipids, cyclodextrins, and glycols, which alter the properties of the mucus, open the junctions between the epithelial cells, and modulate membrane fluidity [63]. It was found that unsaturated fatty acids (e.g., oleic acid, eicosapentaenoic acid or docosahexaenoic acid) significantly enhanced the bioavailability of proteins like insulin [6]. The presence of lecithin increased the thixotropy, viscosity, and the gelation temperature of poloxamer 407 gels [120,121].

Hydroxypropyl β-cyclodextrins (HP β-CDs) are molecular carriers that are used in pharmaceutical formulations, because they increase the stability and solubility of poorly water-soluble drugs, through the formation of inclusion complexes [122]. A system containing both poloxamer 407 and HP β-CDs could be used to combine the peculiar characteristics of the various materials and different experimental investigations have been carried out. HP β-CD_S_ influenced the gelation and micellization temperatures of poloxamer 407-gel and modulated the elasticity of the samples [55]. For example, the addition of different concentrations of HPβ-CDs caused a progressive shift of the gelation temperature of a 20% (*w*/*v*) poloxamer 407 solution towards higher values. The increase of the gelation temperature makes the addition of a greater amount of poloxamer possible: the more elevated the amount of cyclodextrins, the greater the stiffness of the gel will be [55]. Cho et al. developed a new formulation made up of poloxamer 407, HP β-CDs, and chitosan, in order to enhance the permeation of fexofenadine hydrochloride through the nasal epithelium, combining the distinctive aforementioned properties of the different components [106].

In the following sections, the most important recent works concerning the mucosal application of poloxamer 407 gels will be discussed as a function of the administration route.

### 4.1. Rectal Formulations

Rectal formulations are one of the oldest pharmaceutical systems that are used for the treatment of human diseases [123]. Conventional modern suppositories are medicated solid-dosage forms at room temperature, which melt or soften at body temperature [79,124]. The rectal administration of drugs has both systemic and local effects, and is used to treat various diseases, representing a valuable alternative to the oral and parenteral routes [123]. The main advantage of rectal administration is that the drug does not first pass through the liver, as is true with the oral route, thus improving the bioavailability of the active compound; it is also a good alternative for subjects who cannot easily swallow tablets or capsules, such as pediatric and geriatric patients, or for unconscious or uncooperative people. Rectal dosage forms are less painful and more acceptable than injections. Despite this, solid suppositories are not popular due to the resistance of patients which compromises compliance [79].

Thermo-sensitive P407-based hydrogels can be a useful resource for developing “liquid suppositories” that are easily administered in liquid form which become semi-solid systems in situ; this is more acceptable for patients, favoring prolonged drug permanence in the target area [75]. It has been demonstrated that thermo-sensitive in situ gels can increase the bioavailability of nimesulide, ketoprofen, and diclofenac [81,125,126]. A pharmacokinetic study of an in situ gelling-and-mucoadhesive-acetaminophen-liquid suppository made up of poloxamers P407 and P188, and sodium alginate (as mucoadhesive agent), was carried out [94]. In humans, liquid suppositories containing acetaminophen allowed for a faster absorption of the drug, as compared to conventional suppositories, probably because of their better bioadhesive features. In fact, solid-type conventional suppositories are not bioadhesive, and they slowly dissolve and disperse in the rectum, while poloxamer-based formulations rapidly take effect, interacting with the mucosal membranes [94].

Recently, Liu et al. developed a thermo-sensitive in situ gel made up of poloxamer 407, HPMC, and sodium alginate for the rectal delivery of ibuprofen [97]. Ibuprofen is a poorly water-soluble drug, and the dispersion of drug and polymers was the approach used to increase its rectal absorption. HPMC and sodium alginate decreased the T_sol-gel_ of the poloxamer-gel, and increased its strength. This formulation had better drug release than solid suppositories, and it also improved the drug absorption and bioavailability in rabbits [97].

A poloxamer-based liquid suppository was shown to be a suitable carrier, not only for anti-inflammatory or analgesic drugs, but also for other active compounds and vesicular carriers, i.e., levosulpiride, docetaxel, and tizanidine hydrochloride-loaded transfersomes [61,98,99].

### 4.2. Vaginal Formulations

Recently, the vaginal route has been exploited for both systemic and local applications. It offers a favorable alternative to the parenteral route for the delivery of drugs that have important side-effects in the gastrointestinal tract, due to the presence of a dense network of blood vessels, the opportunity to bypass the hepatic first-passage, and its relatively high permeability to a wide range of drugs, such as peptides and proteins [3,127]. Moreover, the vaginal cavity is the site of various viral and bacterial infections that can be successfully treated by a local administration of antiseptic and/or anti-inflammatory drugs [128]. Conventional vaginal medicinal forms include suppositories, films, effervescent tablets, capsules, vaginal rings, and semisolid preparations, such as hydrogels and foams. Due to the ease of administration and their flexibility, semisolid forms such as hydrogels are much more popular than solid formulations [89,129]. However, vaginal drug delivery is often limited because of the protective mechanisms of the vagina, including a wide range of physiological characteristics including pH variation, microflora, and cervical mucus. Particularly in the case of semi-solid formulations, the rapid physiological clearance of a vaginally-administered medication means poor drug retention, leakage, and a reduced duration of the therapeutic effects, all of which make multiple administrations necessary [89,110]. The development of mucoadhesive pharmaceutical formulations that have the ability to efficiently interact with the mucosal tissue of the vagina offers the possibility of prolonging the therapeutic effects of a drug, improving patient compliance, and decreasing the number of administrations necessary [127]. Thermo-sensitive hydrogels characterized by a gelation process that takes place in the vaginal lumen would favor a prolonged residence time of the drugs they contain [129]. They would form a protective layer on the surface of the vaginal mucosa, which is useful in the treatment of local vaginal infections, such as candidiasis, or for contraception through the inhibition of sperm motility [3,127,129].

Timur et al. combined the mucoadhesive properties of chitosan and the thermo-sensitive characteristics of poloxamer 407, in order to develop a thermo-gelling system containing chitosan nanoparticles for the vaginal delivery of tenofovir (TFV), an antiviral agent that is used in the treatment of the human immunodeficiency virus (HIV) [130]. An amount of TFV was entrapped in the gel, with the aim of controlling the leakage of drugs from the nanoparticles. The in vitro analysis evidenced an initial burst release of TFV from the formulation made up of nanoparticles and poloxamer gel, with respect to the gel containing the active compound in the free form and to a solution of the drug. The total amount of TFV released from the nanoparticle/gel system was 85% in 24 h, while that of the drug contained in the poloxamer gel was ~95%. Both of these values were dramatically better than that obtained by the drug solution, which evidenced full leakage of the active compound over a 3 h period. The entrapment of TFV within the nanoparticles retained by the poloxamer-gel had two peculiar features: (1) the burst-release effect, induced by the presence of the free drug within the gel network, and (2) sustained drug leakage for up to 24 h due to the presence of the colloidal systems in the polymeric matrix [130].

The thermo-sensitive characteristics of poloxamer 407 gels have been also used to deliver the potent antifungal drug itraconazole, which is used in the treatment of vaginal candidiasis. This was done with the aim of increasing its therapeutic efficacy because, generally speaking, large dosages of this drug are required, due to its poor solubility and the problems that are associated with oral administration [131,132]. Itraconazole is marketed in two oral formulations, capsules and an emulsion, which are characterized by a bioavailability of approximately 30% and 55%, respectively [132]. Mirza et al. developed a formulation made up of the drug encapsulated within solid lipid nanoparticles (SLNs) dispersed in a poloxamer P407-based gel [110]. They tested three percentages of poloxamer 407 (15%, 18%, 20% *w*/*v*) enriched with carbopol CP 934 for its bioadhesive properties [133]. The three formulations showed a sustained release profile of the drug, but only the 20% version had an ideal gelling temperature (35 °C). The in vivo tests confirmed the bioadhesion of the gel, the absence of irritation, and an increase of its antimicrobial effect with respect to the marketed formulations of the drug [110]. Also in the field of the vaginal delivery of antifungals, Rençber et al. designed a mucoadhesive system containing another antifungal agent, clotrimazole, for the treatment of vaginal infections [111].

In addition to the conventional therapy against pathogens that can normally affect the vaginal environment, poloxamer 407-based gels have also been investigated to treat infertility. Soliman et al. developed and characterized a thermo-sensitive gel containing sildenafil for the treatment of endometrial thinning caused by clomiphene citrate [112]. The latter is used for the induction of ovulation in women with type II eugonadotrophic anovulation, but it exerts negative effects on the endometrium and the cervical mucus, leading to a thinning of the tissue [134]. In the proposed investigation, poloxamer 407 was combined with poloxamer 188, sodium alginate, and hydroxyethylcellulose (HEC), obtaining a thermo-sensible gel that promoted the increase of the pharmacological effect of sildenafil, yet giving rise to no significant side effects [112,135].

### 4.3. Ophthalmic Formulations

Most ocular diseases are treated by the topical application of drugs. Indeed, the blood-aqueous barrier and the blood-retinal barrier prevent the access of systemically administered drugs to the ocular environment [72]. Topical eye drops (formulated as solutions or suspensions) are conventionally preferred due to their convenience, non-invasiveness, and the low manufacturing cost. Unfortunately, they are characterized by a low level of bioavailability and a brief ocular residence time as a consequence of the various physiological barriers present, that is, the conjunctiva, sclera, and cornea as physical barriers, while the blood and lymphatic vessels modulate the drainage of the eye compartment and the clearance of drugs. Moreover, metabolic enzymes such as esterase and carbonic anhydrase metabolize active compounds [136]. A common way of decreasing the drainage rate is to increase the viscosity of the ophthalmic solutions with the aim of moderately prolonging the contact time between the drug and the ocular tissues. Ointments and ocular inserts provide the entrapped active compounds with extended residence time, but they have low patient compliance [93,137]. Recently, in situ gels are being successfully investigated for ocular drug delivery. Particularly, thermo-sensitive systems are better retained in the eye than conventional eye drops, and they are better tolerated by patients. They are dropped as liquid formulations into the eye, and subsequently the phase transition induces the formation of a viscoelastic gel [93,138]. These formulations offer the possibility of applying accurate and reproducible amounts of drugs in liquid form, with respect to gelled formulations that are normally less well tolerated by patients [139]. The use of poloxamer 407 results in clear, colorless, transparent gels. Transparency is a highly desirable characteristic in ophthalmic formulations because non-transparent systems may blur the vision and they are not suitable for patients [140]. Gels containing the co-polymer demonstrated muco-mimetic properties and optical clarity, promoting a significant degree of ocular permeation and an increase in the bioavailability of the drugs [72].

In general, thermo-sensitive poloxamer 407 aqueous solutions lose their gelation ability, being diluted by the lachrymal fluid present in the eye. Higher concentrations of poloxamer can be used in order to contrast this phenomenon, though this approach has a certain irritating effect on the eye [140,141], but the association of poloxamer 407 with other polymers turned out to be a suitable strategy.

Mixtures of poloxamers 407 and 188 are characterized by an ideal gelation temperature, and they can be used for ocular applications. Fathalla et al. investigated the ideal concentration of the two copolymers necessary to obtain a thermo-sensitive system for the ocular delivery of ketorolac tromethamine (KT) [140]. The formulations prepared with 407 and 188-derivatives at a copolymer concentration of 23% and 10% or 15% (*w*/*v*), respectively, demonstrated suitable rheological properties and mucoadhesive characteristics with no conjunctival or corneal irritation. Moreover, in vitro and ex vivo investigations revealed that the KT entrapped in these systems was characterized by a more sustained release as compared to the drug solution [140].

In another investigation, Khan et al. developed a formulation made up of tobramycin sulphate-loaded chitosan microparticles contained in a poloxamer 407/chitosan gel, proposed for the treatment of ocular infections [105]. Carboxymethyl chitosan and gellan gum are other materials combined with poloxamer 407 in order to obtain innovative gels for ophthalmic application [103,104].

### 4.4. Nasal Formulations

In the last decade, nasal drug delivery has been emerging as a potential approach to be used for the topical administration of bioadhesive systems that need to reach the circulatory system and the brain [142]. It is characterized by attractive features such as non-invasiveness, painlessness, improved patient compliance, and the possibility of self-medication [143]. On the other hand, the restricted capacity of the nasal cavity, the scarce permeability of mucosal tissues, and the rapidity of mucociliary clearance are the limiting factors of nasal drug absorption.

In situ gelling systems have emerged as novel formulations useful for effective intranasal drug delivery, because they can be easily administered as solutions and in accurate dosages, they can prolong the residence time of the entrapped drug inside the nasal cavity, and they can improve its bioavailability. Particularly, the thermo-responsive properties of poloxamer 407 have been extensively exploited in the development of in situ nasal gels, in combination with other mucoadhesive compounds [142].

The nasal administration of drugs is the natural choice for the treatment of disorders that directly affect the nasal area, because it has few side effects as compared to the oral and parenteral forms. Antihistamines and corticosteroids are example of compounds that can be used for the cure of rhinosinusitis, decongestion and cold symptoms [144]. For example, mometasone furoate is a potent new corticosteroid that is commercially available as an aqueous suspension (Nasonex^®^-Schering-Plough, Kenilworth, NJ, USA), but its nasal application is limited by rapid elimination brought about by mucociliary clearance [145,146]. In order to avoid the rapid drainage of the formulation from the site, and to obtain a prolonged residence time in the nasal cavity, Altuntaş et al. developed an in situ gel formulation that was made up of poloxamer 407 as thermo-gelling agent, carbopol 974P NF, and polyethylene glycol 400 (PEG400) as a drug-release enhancer [108]. The characterization of the system showed that Carbopol 974P NF significantly decreased the T_sol-gel_ and increased the viscosity as a function of the amount used. Conversely, PEG 400 increased the T_sol-gel_ and decreased the viscosity of the gel. The mucoadhesive strength was mainly dependent on the concentration of the Carbopol 974P NF. The in vitro investigations demonstrated a prolonged release of the drug with respect to the commercial formulation [147].

The intranasal route is a valid alternative to the oral or parenteral ways for the administration of peptides and proteins because it avoids the hepatic first-passage effect [107,148]. Mura et al. described a nasal delivery system of opiorphin (OPI), characterized by a liposomal formulation contained in a thermo-sensitive poloxamer 407 hydrogel [107]. OPI is a natural peptide that has recently been isolated from human saliva. It appears to be an interesting and promising therapeutic agent in the treatment of acute and chronic pain, due to its strong analgesic effect, which is similar to that of morphine, but without its side effects. Its short pharmacological effect upon intravenous administration is caused by its rapid degradation by the peptidases present in the bloodstream, which limits the clinical application of this peptide [149,150,151]. However, the encapsulation of the compound in the liposomal vesicles dispersed in the poloxamer gel promoted the nasal residence time of the peptide and modulated its release. In detail, the formulation made up of poloxamer 407 (26.5% *w*/*v*) and Carbopol 934P (1% *w*/*v*) evidenced the best properties in terms of proper gelation time, adequate mucoadhesion and gel strength; the presence of liposomes did not influence the rheological features of the system. The ex vivo permeation experiments carried out on excised porcine nasal mucosa revealed that the hydrogel formulation induced a prolonged delivery of the drug for up to 5 h, and demonstrated the essential role of vesicles in the increase of the permeation profile of the compound through the nasal mucosa [107].

The existence of a direct connection between the olfactory region of the nasal cavity and the cerebral-spinal fluid was used by the scientific community to investigate the opportunities for exploiting the nasal mucosa for the delivery of drugs to the brain [152]. Intranasal drug delivery is an alternative to invasive approaches, such as intra-cerebroventricular or intra-parenchymal injections of molecules, and it is done to bypass the blood brain barrier (BBB), which limits the passage of drugs from the blood into the central nervous system (CNS) [143,153]. “Nose to brain” delivery is a suitable option for reaching the brain, but it is only efficacious when the drug remains in contact with the nasal epithelium long enough to diffuse into the olfactory projections, avoiding clearance by mucus and ciliary movement [109]. For this reason, poloxamer hydrogels were used as potential formulations that are able to promote the “nose to brain” delivery of many active compounds (i.e., levodopa, selegiline, anti-Parkinson’s drugs, etc.) thanks to their peculiar mucoadhesive features that are previously described [109,154,155,156,157,158,159,160].

### 4.5. Buccal Formulations

The buccal administration of drugs offers several advantages, due to the good vascularity of the mouth, the consequent bioavailability, plus the opportunity of bypassing the hepatic first-passage and gastrointestinal degradation [161]. However, the accidental swallowing of the formulations besides continuous dilution by saliva allow for only a short drug residence time in the oral cavity [162]. The mucoadhesive poloxamer 407-based gel systems were also used in this case to establish strong contact with the buccal mucosa, increasing the residence time of the contained drugs and improving their bioavailability [163,164,165]. For example, Nasra et al. developed a poloxamer 407-based hydrogel for the buccal delivery of curcumin, with the aim of exploiting the anti-inflammatory properties of the substance to decrease the inflammatory mediators involved in periodontitis [164]. Carbopol P 934 was added as a pH-sensitive and mucoadhesive agent, and the obtained formulation was easily syringeable and characterized by a suitable gelation temperature and viscosity. The stability of the curcumin was maintained and the formulations demonstrated that they were clinically efficacious in terms of the reduction of the probing depth, bleeding index and amount of plaque [164].

In another investigation, Sheshala et al. developed and characterized a thermo-sensitive mucoadhesive gel made up of poloxamer 407 (21% *w*/*v*), poloxamer 188 (2% *w*/*v*), and HPMC (0.5% *w*/*v*) for the buccal delivery of the antibiotic moxifloxacin [163]. The formulation brought about a constant and sustained drug release with an initial burst effect of 8 h, and demonstrated a promising antimicrobial efficacy against *Aggregatibacter Actinomycetemcomitans* and *Streptococcus mutans*, bacteria, which are responsible for various periodontal infections [163].

## 5. Conclusions

Thanks to their particular thermo-reversible and promising drug-release properties, poloxamer 407-based hydrogels are attractive pharmaceutical formulations that can be administered to patients by various routes. Due to their liquid state before administration at room temperature, they are easy to manage during the manufacturing process, and administration and improve the compliance of patients. The subsequent sol–gel transition, combined with the action of the mucoadhesive polymers that make up the hydrogel, establishes a prolonged and sustained drug release of the entrapped compounds, decreasing their efficacious dosage, side effects, and the number of necessary administrations. At the best of our knowledge, only a gel formulation containing poloxamer 407 (20% *w*/*v*), named LeGoo^®^ (developed by Pluromed Inc., Woburn, MA, USA), was approved by the U.S. FDA for temporary endovascular occlusion of blood vessels below the neck up to 4 mm in diameter (https://www.accessdata.fda.gov/scripts/cdrh/cfdocs/cfpma/pma.cfm?ID=P110003).

Unfortunately, no type of other systemic and local administration of poloxamer 407-based hydrogels has been approved for clinical use up to now, probably due to various sticking points in clinical translation with regards to both delivery aspects (e.g., biological challenges) and regulatory aspects (e.g., study design and approval challenges). In fact, there is a general lack of regulatory standards with regard to the examination of supramolecular-based therapeutics, so significant efforts are being made in this direction [166]. Moreover, the lipidic profile alteration, the possible renal toxicity, and the immunomodulation exerted by poloxamer 407 after parenteral administration are detrimental for a significant translation of this material in clinical practice [6]. Much more research and human clinical trials will be necessary in order to evaluate the benefits-to-risk ratio of poloxamer 407-based hydrogels.

## Figures and Tables

**Table 1:** Examples of P407-based mucoadhesive hydrogels and their potential applications.

Pharmaceutical Formulations	%P407 (/P188) ^1^	Additives	Results	Reference
**Rectal application**				
Rectal administration of acetaminophen formulated as a liquid suppository.	15/15 15/20	PVP ^2^, HPMC ^3^, HPC ^4^, Carbopol 934P, Polycarbophil	PVP, HPMC, and HPC: non-affected T_sol-gel_. Carbopol and polycabophil decreased T_sol-gel_, and enhanced the gel strength and bioadhesive force.	[79]
P407/P188 liquid suppository bases	15/15	Ethanol, propylene glycol, glycerin, hydrochloric acid, sodium chloride, sodium monohydrogen phosphate, sodium dihydrogen phosphate	Sodium chloride, sodium monohydrogen phosphate, and sodium dihydrogen phosphate increased the gel strength and the bioadhesive force, with a decrease in gelation temperature. Glycerin slightly decreased the gelation temperature, and slightly increased the gel strength and bioadhesive force.	[76]
Propranolol mucoadhesive liquid suppositories.	15/15	HPC, PVP, Carbopol, sodium alginate, polycarbophil	Sodium alginate exhibited the greatest degree of mucoadhesion and caused no irritation of the rectal mucosal membrane.	[95]
Thermo-sensitive and mucoadhesive rectal in situ gel of nimesulide.	18	Sodium alginate, HPMC, polyethylene glycol (PEG 4000 and PEG 400)	The addition of PEG polymers increased the gelation temperature and the drug release rate. The P407/nimesulide/sodium alginate/PEG 4000 (18/2.0/0.5/1.2%) exhibited the appropriate gelation temperature, acceptable drug release rate, and rectal retention.	[81]
Thermo-sensitive gels based on poloxamer 407 and HPMC for the rectal delivery of quinine for the treatment of severe malaria in children.	16, 17	Propanediol-1,2, HPMC	1,2-Propanediol limits HPMC precipitation in poloxamer 407 solution. Moreover, HPMC in the presence of propanediol-1,2 had a synergistic effect on the gelation of the poloxamer 407 solution.	[75]
Thermo-sensitive poloxamer gel containing diclofenac sodium in a rectal dosage form.	15/17	Sodium chloride	Rectal diclofenac sodium/P407/P188/sodium chloride gel could provide fast drug absorption, without damaging the rectum.	[96]
In situ gelling and mucoadhesive acetaminophen liquid suppository.	15/19	Sodium alginate	Acetaminophen liquid suppository allowed faster absorption of acetaminophen in human subjects than conventional suppositories, probably because of its greater dispersability and bioadhesive force.	[94]
Thermo-sensitive in situ gel based on solid dispersions for rectal delivery of ibuprofen.	20	HPMC, sodium alginate	HPMC and sodium alginate lowered T_sol-gel_ and increased gel strength. Liquid suppository showed better drug release performance than solid suppositories, and the drug absorption and bioavailability were both improved in rabbits.	[97]
Levosulpiride-loaded liquid suppositories with improved bioavailability.	15/17	Tween 80	Tween 80 increased the mucoadhesive force and the gel strength. The system showed a suitable gelation temperature and exhibited an enhanced bioavailability with respect to the drug suspension in rats.	[61]
Nanotransfersome-loaded thermosensitve in situ gel as a rectal delivery system of tizanidine.	21/3	HPMC	An increase in the bioavailability and a sustained release of the drug were obtained by the synergic effect of a poloxamer gel and nanotransfersomes.	[98]
Docetaxel-loaded thermo-sensitive liquid suppository	11/15	Tween 80	Tween 80 induced an increase in viscosity.	[99]
**Ophthalmic application**				
Thermo-reversible in situ gelling ophthalmic drug delivery system based on Pluronic F 127, containing moxifloxacin hydrochloride	15 (*w*/*v*)	Gelrite^®^	Gelrite^®^ showed a positive effect on the bioadhesive features.	[100]
P407/chitosan ophthalmic delivery system characterized by a prolonged retention time for the treatment of ocular diseases.	14–20	Chitosan	Chitosan improved the mechanical strength and textural properties of poloxamer formulations, characterized by a significant residence time in the eye.	[101]
Alginate and Pluronic-based in situ gelling system for ophthalmic delivery of pilocarpine.	12–16	Alginic acid	The rheological analysis as well as in vitro and in vivo studies demonstrated that the alginate/Pluronic mixture was used to retain pilocarpine in order to increase its ocular bioavailability.	[102]
Poloxamers/ hyaluronic acid (HA) gel for the ocular delivery of acyclovir	15/10, 15	Hyaluronic acid	The addition of HA caused a modulation in the rheological properties of the poloxamer. Mucoadhesion tests showed an increased interaction with mucin. In vitro analysis showed a controlled release of acyclovir.	[80]
A dual pH- and temperature-responsive poloxamer 407-hydrogel system containing carboxymethyl chitosan cross-linked by glutaraldehyde for ophthalmic drug delivery.	1.5–20 (*w*/*v*)	Carboxymethyl chitosan	No toxicity on human corneal epithelial cells at a low concentration. The gelation temperature was 32–33 °C, suitable for ocular delivery, while the viscosity quickly increased after gelation, and a sustained release of the drug was observed.	[103]
Combined poloxamer 407/gellan gum in situ gel for the ocular delivery of pilocarpine hydrochloride	18	Gellan gum	Gellan gum caused a decrease in the gelation temperature and an increase of viscosity due to the formation of hydrogen bonds with the poloxamer. In addition, gellan gum largely decreased the gel dissolution rate, while in an vitro drug release study, it showed a better drug delivery time with respect to the poloxamer alone.	[104]
Tobramycin sulfate-loaded microparticles dispersed in poloxamer 407/chitosan thermosensitive gel for the treatment of ocular infections	17 (*w*/*v*)	Chitosan	Addition of chitosan resulted in an increase in viscosity and in a greater mucoadhesive strength of the gel. It also evidenced a better in vitro permeability and a greater aqueous humor concentration of the drug, compared with commercial tobramycin eye drops with no signs of ocular irritation.	[105]
**Nasal application**				
Poloxamer/cyclodextrin/chitosan-based thermoreversible gel for the intranasal delivery of fexofenadine hydrochloride.	17 (*w*/*v*)	Chitosan	Chitosan induced a slight increase in gelation temperature and viscosity, promoting a controlled release of the drug and a significant permeation through the nasal epithelium.	[106]
In situ mucoadhesive-thermosensitive liposomal gel as a novel formulation for the nasal delivery of opiorphin	15–30 (*w*/*v*)	Carbopol 934P, HPMC, P188	The formulation made up of poloxamer 407 (26.5% *w*/*v*) and carbopol 934P (1% *w*/*v*) showed the best properties in terms of proper gelation time, adequate mucoadhesive and gel strength, and mucoadhesion duration. This hydrogel had a prolonged, controlled delivery of the drug for more than 5 h, and the liposomes enhanced the permeability coefficient and the permeation rate of the peptide up to six times.	[107]
Thermo-reversible in situ nasal gels containing mometasone furoate for the treatment of allergic rhinitis	18	Carbopol 974P, PEG 400	Carbopol 974P NF significantly decreased the T_sol-gel_ and increased the viscosity, while PEG 400 increased the T_sol-gel_ and decreased gel viscosity. Mucoadhesive strength was predominantly dependent on the Carbopol 974P NF. The release of the drug was prolonged, as demonstrated by in vitro experiments.	[108]
Mucoadhesive thermo-sensitive nasal gel of selegiline hydrochloride for the treatment of Parkinson’s disease.	15–18	Chitosan	The formulation showed desired characteristics such as sol–gel transition at nasal temperature, viscosity, pH, and mucoadhesive strength, and it improved the drug residence time in the nasal cavity. In vivo investigations confirmed that selegiline hydrochloride was more efficacious after encapsulation within the thermo-sensitive gel with respect to the nasal solution or oral tablets.	[109]
**Vaginal application**				
Thermo-sensitive and mucoadhesive vaginal gel containing clotrimazole	15/15, 20	Polycarbophil	The formulation had a useful gelation time and T_sol-gel_ values, as well as suitable rheological properties, even after dilution with simulated vaginal fluid.	[89]
Mucoadhesive and thermo-sensitive poloxamer 407-based gel for the topical delivery of itraconazole	15, 18, 20 (*w*/*v*)	Carbopol CP 934	The gel demonstrated an appreciable bioadhesion and non-toxicity. A remarkable decrease in the microbial count was observed, as compared to the marketed formulation.	[110]
Vaginal mucoadhesive in situ gel formulations of clotrimazole	20/10	HPMC E50, HPMC K100M	The rheological and texture analysis revealed a suitable gelation temperature and time, together with an appropriate consistency, high adhesiveness, cohesiveness, and mucoadhesiveness values. In vivo studies showed a long residence time in the vaginal compartment (up to 24 h).	[111]
In situ thermo-sensitive gels for the vaginal administration of sildenafil as a potential treatment of infertility in women.	15–20/15, 20	Sodium alginate, HEC ^5^	P188 increased the T_sol-gel_ and mucoadhesive force. HEC and sodium alginate increased the viscosity and the mucoadhesion. All polymers showed a significant decrease of released sildenafil. Clinical results showed that the vaginal gel containing sildenafil significantly increased endometrial thickness and the uterine blood flow with no side effects.	[112]

^1^ each value is expressed as % *w*/*w* concentration, when not specified; ^2^ polyvinylpyrrolidone; ^3^ hydroxypropylmethylcellulose; ^4^ hydroxypropylcellulose; ^5^ hydroxyethylcellulose.
